# Plant genome editing with TALEN and CRISPR

**DOI:** 10.1186/s13578-017-0148-4

**Published:** 2017-04-24

**Authors:** Aimee Malzahn, Levi Lowder, Yiping Qi

**Affiliations:** 1 Department of Plant Science and Landscape Architecture, University of Maryland, College Park, MD 20742 USA; 2 Department of Biology, East Carolina University, Greenville, NC 27858 USA; 3 Institute for Bioscience and Biotechnology Research, University of Maryland, Rockville, MD 20850 USA

**Keywords:** Plant genome editing, TALEN, CRISPR, Cas9, Cpf1, NHEJ, HDR

## Abstract

Genome editing promises giant leaps forward in advancing biotechnology, agriculture, and basic research. The process relies on the use of sequence specific nucleases (SSNs) to make DNA double stranded breaks at user defined genomic loci, which are subsequently repaired by two main DNA repair pathways: non-homologous end joining (NHEJ) and homology directed repair (HDR). NHEJ can result in frameshift mutations that often create genetic knockouts. These knockout lines are useful for functional and reverse genetic studies but also have applications in agriculture. HDR has a variety of applications as it can be used for gene replacement, gene stacking, and for creating various fusion proteins. In recent years, transcription activator-like effector nucleases and clustered regularly interspaced palindromic repeats (CRISPR) and CRISPR associated protein 9 or CRISPR from Prevotella and Francisella 1 have emerged as the preferred SSNs for research purposes. Here, we review their applications in plant research, discuss current limitations, and predict future research directions in plant genome editing.

## Background

The field of genome editing is experiencing rapid growth as new methods and technologies continue to emerge. Using genome editing to boost agriculture productivity is needed as the world population is expected to grow to 9.6 billion by 2050 while the amount of arable land decreases [1]. Besides potential for boosting crop yields, genome editing is now one of the best tools for carrying out reverse genetics and is emerging as an especially versatile tool for studying basic biology.

Genome edited plants are differentiated from conventional transgenic plants as they may not incorporate foreign DNA. Although genome editing can be used to introduce foreign DNA into the genome, it may simply involve changes of a few base pairs in the plant’s own DNA. This distinction makes genome editing a novel and powerful breeding tool that has promising applications in agriculture, especially when genome edited crops are not regulated as genetically modified (GM) [2].

## Genome editing relies on DNA repair

**Fig. 1:**
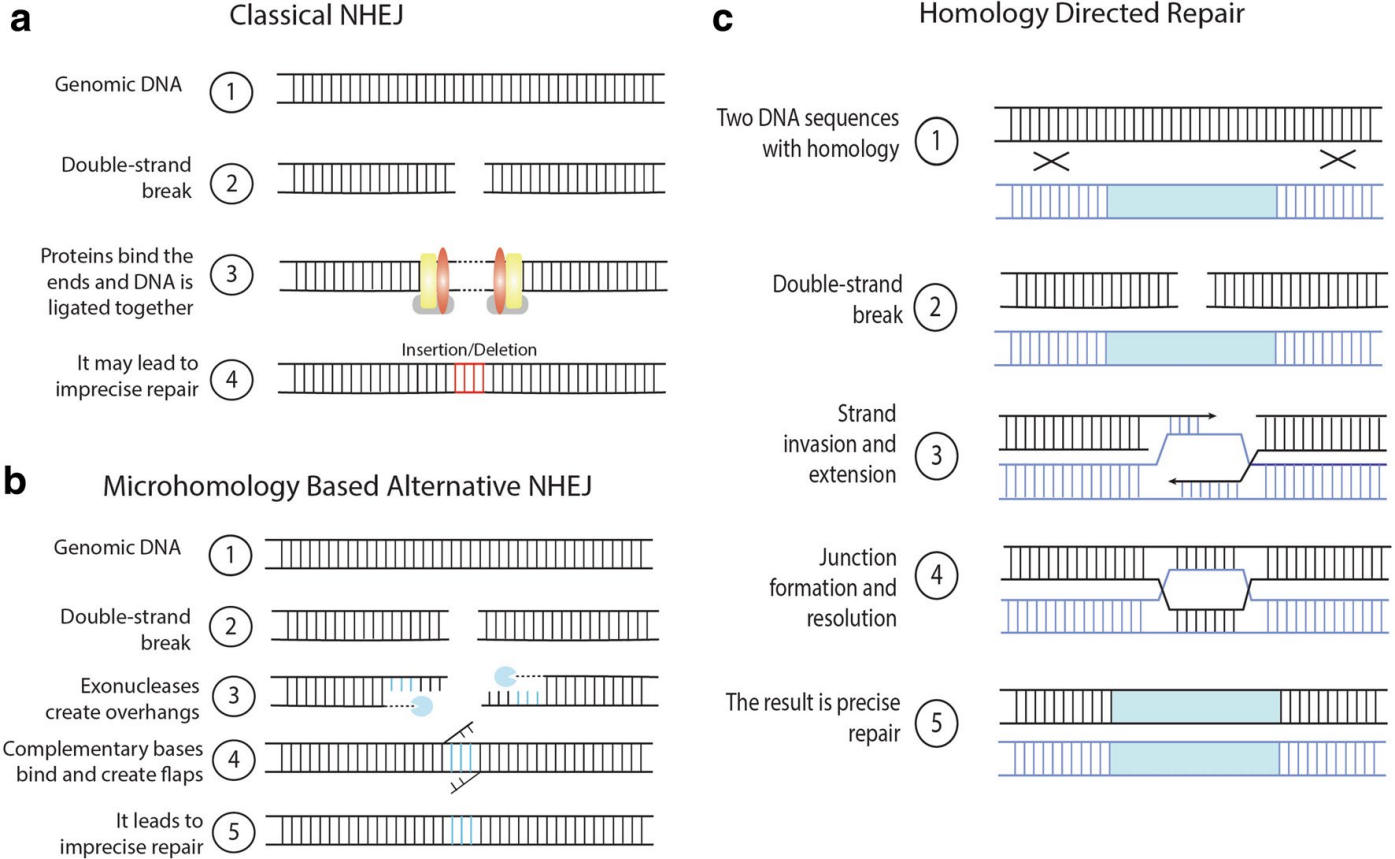
Major DNA repair pathways in plants. Non-homologous end joining (NHEJ) and homology directed repair (HDR) are two main repair pathways. Classical NHEJ may lead to insertions or deletions, while microhomology based alternative NHEJ always results in deletions. Homology directed repair is less efficient, but can result in precise integration of a donor DNA template into the genome

DNA damage occurs naturally in all cells either due to exogenous factors, such as UV radiation, or endogenous agents such as metabolic by-products and free radicals. A double-strand break (DSB) is the most lethal type of DNA damage and must be repaired before DNA replication, which has led to the evolution of two major DNA repair pathways in eukaryotes: non-homologous end-joining and homology-directed repair [3–6] (Fig. 1).

Non-homologous end-joining (NHEJ) is an error-prone repair pathway. When a DSB occurs, NHEJ can quickly, although often imprecisely, be used in two ways to repair the break. In classical NHEJ (Fig. 1a), several different proteins (e.g. Ku70 and Ku80) bind to broken DNA ends and are joined together by a ligase that can result in the insertion or deletion (indel) of nucleotides. In microhomology-based alternative NHEJ (Fig. 1b), 5′ ends are cut until 3′ overhangs with homology are created. DNA strands then bind at their complementary sequence, and flaps of non-homologous DNA are excised. This typically results in deletions as DNA between homologous sections are removed. NHEJ often leads to frameshift mutations which can result in premature stop codons, rendering genes non-functional (Fig. 1a, b). This is helpful for creating knockout plants useful for reverse genetic studies, but can also create desirable agricultural traits. For example, a powdery mildew resistant wheat line was created by knocking out three redundant *MLO* genes [7].

The second DNA repair pathway is homology directed repair (HDR) which relies on template DNA. Homologous recombination is an important process that occurs in somatic cells to repair DSBs and in meiotically dividing cells to exchange genetic material between parental chromosomes. The most common conservative HDR mechanism in plants, which repairs almost all DSBs in somatic cells, is the synthesis-dependent strand annealing (SDSA) pathway [4, 8] (Fig. 1c). As a DSB occurs, 3′ overhangs are extended from the break site. A 5′ end invades the homologous strand forming a D-loop. Synthesis fills in the gaps using homologous DNA as a template, and the 3′ end reanneals with the second 3′ end without crossover. The result is a precisely integrated template or “donor” DNA strand. In nature, template DNA in the form of a sister chromatid or homologous chromosome is not always available, which may hinder HDR. However, synthetic template DNA can be provided exogenously and used for gene insertion, replacement, or epitope/florescent tagging. There are many exciting applications in basic and applied science using HDR. For example, HDR was used to engineer an herbicide resistant trait in tobacco plants [9].

## Rapid evolution of sequence specific nucleases (SSNs) for plant genome editing

Meganucleases, or homing endonucleases, are site specific endonucleases found in eukaryotes, archaea, and bacteria which recognize DNA sequences over 12 bp long [10]. Several hundred meganucleases have been discovered and they can be divided into four families: LAGLIDADG, His-Cys box, GIY-YIG, and the HNH family [10]. The LAGLIDADG family consists of popular meganucleases I-CreI and I-SceI. Originally, meganucleases were only able to target a single sequence and thus were not capable of targeting endogenous genes. After it was discovered that only a few amino acid residues make direct contact with nucleotides, the binding specificity was successfully altered for targeting endogenous genes. For example, targeted mutagenesis was successfully achieved in maize with de novo-engineered meganucleases [11]. However, DNA binding properties of meganucleases cannot be completely separated from their nuclease activity, making them difficult to engineer and use in research.

Zinc finger nucleases (ZFNs) function as dimers and each monomer is a fusion protein of a zinc finger DNA binding domain and a non-specific FokI nuclease domain [12, 13]. A zinc finger is formed by repeated groupings of cysteine and histidine residues and recognize 3 nucleotides (nt). Each ZFN monomer is typically composed of 3 or 4 zinc fingers, recognizing 9 or 12 nt DNA. The zinc fingers are thought to be modular, making it possible to recognize a long stretch of DNA by putting multiple zinc fingers together [14, 15]. However, ZFNs based on modular assembly typically have poor activity and high toxicity [16, 17], suggesting there is context dependency among neighboring fingers. This context dependency in ZFN engineering has been largely addressed by a proprietary platform developed by Sangamo Bioscience [18] and by academically developed platforms such as “OPEN” [19] and “CoDA” [20]. “OPEN” or “CoDA” generated ZFNs were later used for generating mutants and studying DNA repair mechanisms in the model plant Arabidopsis [21–23].

**Fig. 2:**
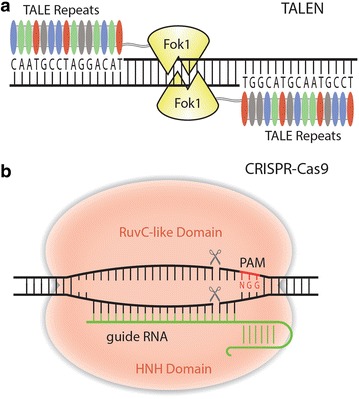
TALEN and CRISPR-Cas9. **a** A TALEN is composed of two monomers with each containing a TALE DNA binding domain and a FokI nuclease domain. Fok1 dimerizes to create a double-strand break. **b** CRISPR-Cas9 is a two-component system composed of Cas9 and a gRNA. Once Cas9 finds a PAM site, if the gRNA binds to the DNA, a double break occurs three base pairs upstream the PAM

The possibility of engineering transcription activator-like (TAL) effectors for DNA targeting was realized in 2009 when their DNA binding mechanism was discovered [24, 25]. TAL effectors in nature are introduced into plant host cells by the bacterium *Xanthomonas* via the type III secretion system, where they alter host gene expression to meet the bacteria’s needs. In the nucleus, TAL effectors bind target genes’ promoters within 60 base pairs of start codons and activate transcription [24]. The DNA binding central repeat domain of each TAL effector is composed of a few to 33.5 repeats which are typically made of 34 amino acids [26]. Using a β-glucuronidase (GUS) reporter in tobacco, Boch et al. discovered repeat variable diresidue (RVD) at positions 12 and 13 of each repeat determines nucleotide binding specificity [25]. This breakthrough quickly led to the creation of a new kind of SSN called TAL effector nuclease (TALEN), which is based on the fusion of a Fok1 nuclease domain to the DNA binding TALE repeats [27–30] (Fig. 2a). There are benefits to choosing TALENs over ZFNs. First, TALEs are less toxic and secondly, they are easier to engineer because recognizing each DNA nucleotide simply relies on using a TALE repeat with the corresponding RVD. However, the repetitive sequence of TALE makes them difficult to construct via polymerase chain reaction (PCR). This was addressed with the development of multiple assembly methods mostly based on Golden gate cloning (e.g. [31–33]), which furthered rapid adoption of TALEN technology for genome editing in many organisms including plants.

Just 2 years after the realization of TALENs, another genome editing tool was introduced. Clustered regularly interspaced palindromic repeats (CRISPR) had been found to function as an adaptive immune system in bacteria and archaea against invading viruses, phages and plasmids [34–36]. The bacteria can protect themselves using a series of CRISPR associated (Cas) proteins that cleave viral DNA, insert pieces of viral DNA into their own genomes, and then use certain Cas9 protein(s) paired with RNA transcribed from the viral DNA library to make targeted double-strand breaks in invading viral DNA. Class 2 CRISPR-Cas systems utilize single-protein effectors, such as Cas9, for DNA targeting [37]. Cas9 is composed of two endonuclease domains, HNH and a RuvC-like domain that each cut one strand of DNA (Fig. 2b). It was demonstrated in 2012 that Cas9 of *Streptococcus pyogenes* could be paired with a synthetic single guide RNA (gRNA) to create a targeted DNA DSB in vitro and in *Escherichia coli* [38]. Shortly after, CRISPR-Cas9 was demonstrated as a powerful RNA-guided SSN for genome editing in human cells [39, 40]. Although off target effects have been a concern, the simple design and ease of vector construction has dramatically increased the number of genome editing studies using CRISPR-Cas9 in plants [41, 42].

**Table 1:** Comparison of TALEN and CRISPR-Cas9 systems

*TALEN*	
Advantages	Disadvantages
~30 bp target requirement results in less off-target effects	Difficult protein engineering potentially increases time and financial investment
No PAM requirement; can target any sequence	Efficiency varies for each construct
	Cannot target methylated DNA
	Difficult to engineer nickase
*CRISPR-Cas9*	
Advantages	Disadvantages
Able to multiplex	Higher potential for off-target effects
Easy to engineer	PAM requirement limits target
Can target methylated DNA	
Easy to create a nickase	

Both TALEN and CRISPR-Cas9 have been used extensively for genome editing and each have their own unique disadvantages and advantages (Table 1), that will be further explored in this review. Both systems will continue to be useful as molecular scissors for a wide variety of applications.

## NHEJ based genome editing by TALEN

Over 50 genes have been targeted for mutations using TALEN in plants, including Arabidopsis, Barley, Brachypodium, maize, tobacco, rice, soybean, tomato and wheat (Table 2). Many of these have been proof-of-concept studies. TALEN scaffolds were optimized for high activity in plants [43]. The optimized TALEN scaffold was then demonstrated by targeted mutagenesis in Arabidopsis [44], tomato [45], Brachypodium [46] and wheat [7]. More recently, TALEN was shown to induce a variety of heritable mutations in rice [47], demonstrating its usefulness in plant genome editing.

**Table 2:** TALEN mediated genome editing in plants

Plant species	Target gene	Modification	Reference
Arabidopsis	*ADH1, TT4, MAPKKK1, DSK2B, NATA2, GLL22a, GLL22b*	NHEJ	[31, 44]
Arabidopsis	*CLV3*	NHEJ	[134]
Arabidopsis	*CRU3*	NHEJ	[135]
Barley	*HvPAPhy_a*	NHEJ	[136]
Barley	*GFP (transgene)*	NHEJ	[137]
Barley	*GFP (transgene)*	HDR	[92]
Brachypodium	*ABA1, CKX2, SMC6, SPL, SBP, COlI, RHT, HTA1*	NHEJ	[46]
Maize	*GL2*	NHEJ	[138]
Maize	*IPK1A, IPK, MRP4*	NHEJ	[139]
*Nicotiana benthamiana*	*FucT, XylT*	NHEJ	[140]
*Nicotiana tabacum*	*ALS*	NHEJ, HDR	[43]
Potato	*Vlnv*	NHEJ	[52]
Potato	*ALS*	NHEJ	[141, 142]
Rice	*11N3*	NHEJ	[48]
Rice	*DEP1, BADH2, CKX2, SD1*	NHEJ	[46, 50]
Rice	*EPSPS*	NHEJ	[143]
Rice	*MST7, MST8, PMS3, CSA, DERF1*	NHEJ	[47]
Rice	*LOX3*	NHEJ	[51]
Rice	*ALS*	HDR	[93]
Rice	*SWEET14*	NHEJ	[144]
Rice	*WAXY*	NHEJ	[145]
Soybean	*FAD2*-*1A, FAD2*-*1B, FAD3A*	NHEJ	[49, 146]
Soybean	*PDS11, PDS18*	NHEJ	[147]
Sugarcane	*COMT*	NHEJ	[148]
Tomato	*PROCERA*	NHEJ	[45]
Tomato	*ANT1*	HDR	[94]
Wheat	*MLO*	NHEJ	[7]

As an effective genome editing tool, TALEN has been applied to generate useful traits in crops. In an elegant study, TALEN was used to engineer disease resistance in *Xanthomonas oryzae* pv. *oryzae* by destroying the target sequence of TALE effectors in rice [48]. In soybean, the *FAD2* gene was targeted for improved oil quality [49]. In wheat, three homologs of *MLO* were successfully targeted for simultaneous knockout, conferring heritable disease resistance to powdery mildew [7]. Improved rice seeds have been engineered with TALEN, creating traits such as fragrance [50] and storage tolerance [51]. Improved cold storage and processing traits have also been engineered in potato [52].

**Fig. 3:**
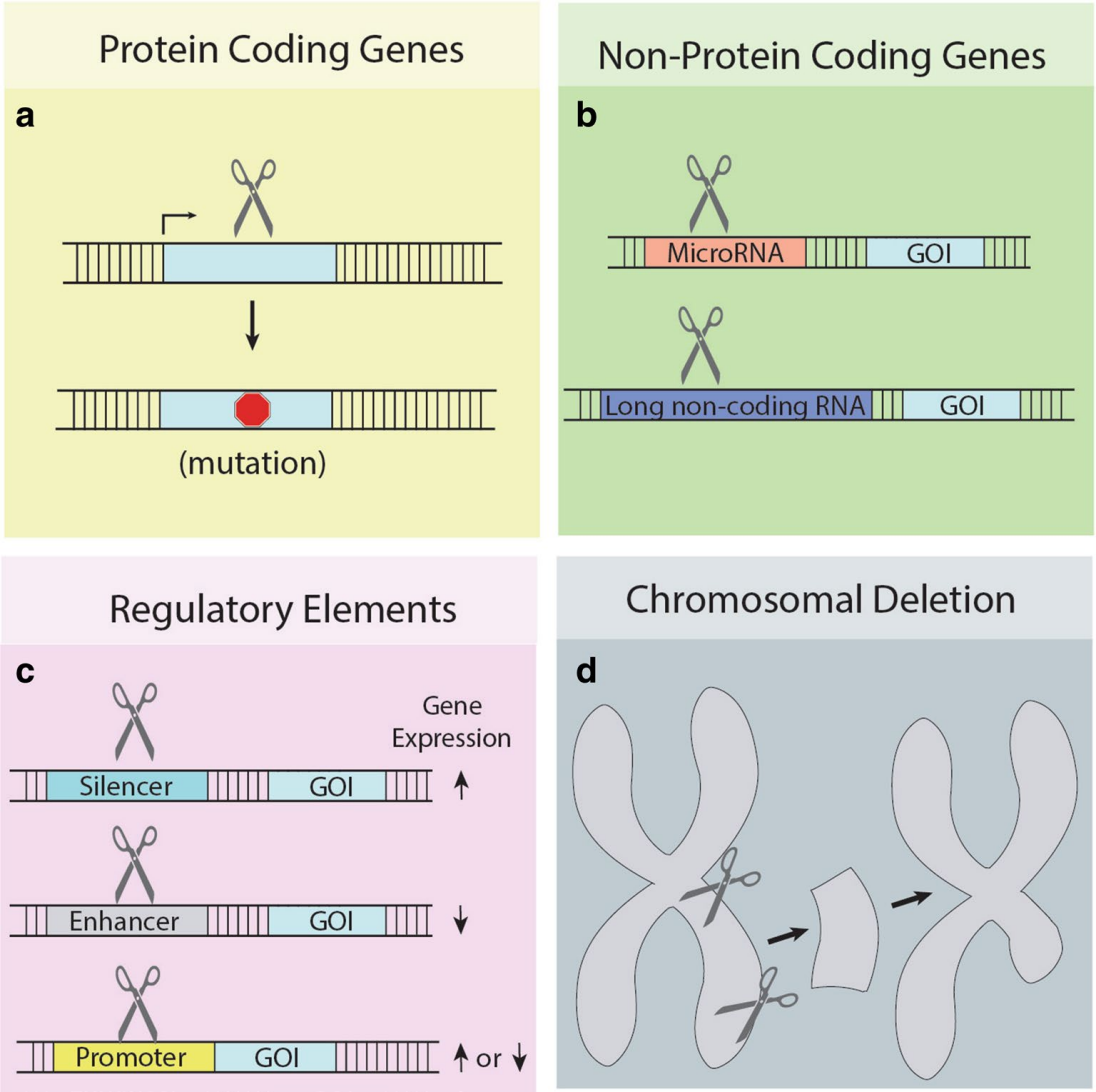
NHEJ based genome editing applications. **a** NHEJ repair of an SSN induced break can create a premature stop codon. A stop codon is indicated by a red octagon. GOI is an acronym for gene of interest. **b** Non-protein coding genes such as microRNA and long non-coding RNA can be rendered non-functional through targeted mutations by SSNs. **c** Regulatory elements involved in the activation or repression of genes can be disrupted by SSNs. **d** Pieces of chromosomes that may involve regulatory networks or related genes can be deleted by SSNs

Most of these studies targeted protein coding genes for mutagenesis (Fig. 3a). Other types of NHEJ based editing can also be achieved by TALEN, such as targeted mutagenesis of non-protein coding genes (Fig. 3b) and regulatory elements [48] (Fig. 3c), and generating large chromosomal deletions [44] (Fig. 3d).

## NHEJ based genome editing by CRISPR-Cas9

Due to ease of engineering, CRISPR-Cas9 has been widely adopted for genome editing in plants (Table 3). At the time of this review, CRISPR-Cas9 edited plants include Arabidopsis, barley, *Brassica oleracea*, cotton, dandelion, flax, lettuce, liverwort, corn, petunia, populus, rice, sorghum, soybean, sweet orange, tomato, wheat, and several tobacco varieties (Table 3). CRISPR-Cas9 quickly moved beyond proof-of-concept; promoting a reverse genetics revolution in plant research and creating many desirable traits in major crops. Using rice as an example, multiple yield-related genes have been targeted in rice [53]. CRISPR-Cas9 has been widely used for functional study on rice genes (Table 3). In addition, environment-induced male sterility has been engineered to facilitate hybrid-based breeding [54, 55]. Disease resistance traits have been developed by knocking out host genes in rice [56] and Arabidopsis [57].

**Table 3:** CRISPR-Cas9 mediated genome editing in plants

Plant species	Target gene	Modification	Reference
Arabidopsis	*PDS3, FLS2, RACK1b, RACK1c*	NHEJ	[96]
Arabidopsis	*BRI1, GAI, JAZ1*	NHEJ	[149, 150]
Arabidopsis	*CHLI1, CHLI2, TT4, AP1, GL2*	NHEJ	[150–152]
Arabidopsis	*GFP (transgene)*	NHEJ	[153, 154]
Arabidopsis	*ADH1, TT4, RTEL, GUS (transgene)*	NHEJ, HDR	[85, 86]
Arabidopsis	*FT, SPL4*	NHEJ	[155]
Arabidopsis	*ABP1*	NHEJ	[71]
Arabidopsis	*Cru3*	NHEJ	[156]
Arabidopsis	*TRY, CPC, ETC2, CHIL1, CHIL2*	NHEJ	[79, 157]
Arabidopsis	*1g03180, 1g16210, 1g56650, 5g55580*	NHEJ	[80]
Arabidopsis	*05g55580, 1g56650, 1g03180, 1g16210*	NHEJ	[80]
Arabidopsis	*PHYB, BRI1*	NHEJ	[123]
Arabidopsis	*BRI1, PDS3*	NHEJ	[158]
Arabidopsis	*PYR1, PYL1, PYL2, PYL4, PYL5, PYL8*	NHEJ	[82]
Arabidopsis	*SH3P3*	NHEJ	[159]
Arabidopsis	*eIF(iso)4E*	NHEJ	[57]
Arabidopsis	*CBF1, CBF2, CBF3*	NHEJ	[63, 160]
Arabidopsis	*DM2*	NHEJ	[161]
Arabidopsis	*UGT79B2, UGT79B3*	NHEJ	[162]
Arabidopsis	*CWIN1*	NHEJ	[163]
Arabidopsis	*MIR169a, MIR827a, TFL1*	NHEJ, HDR	[77]
Arabidopsis	*TTG1*	NHEJ	[164]
Barley	*HvPM19*	NHEJ	[165]
Cabbage	*BoIC.GA4.a*	NHEJ	[165]
Camelina	*FAD2*	NHEJ	[69, 70]
*C. reinhardtii*	*CpFTSY, ZEP*	NHEJ	[166]
Cotton	*GFP (transgene)*	NHEJ	[167]
Cotton	*MYB25*-*like A, MYB25*-*like D*	NHEJ	[67]
Cotton	*CLA1, VP*	NHEJ	[68]
Dandelion	*1*-*FFT*	NHEJ	[168]
Flax	*EPSPS, BFP (transgene)*	NHEJ, HDR	[169]
Grape	*IdnDH*	NHEJ	[170]
Lettuce	*BIN2*	NHEJ	[123]
Liverwort	*ARF1*	NHEJ	[171]
*Lotus japonicus*	*SYMRK, LjLb1, LjLb2, LjLb3*	NHEJ	[172]
Maize	*IPK*	NHEJ	[139]
Maize	*LIG1, Ms26, Ms45, ALS1, ALS2*	NHEJ, HDR	[173]
Maize	*PSY1, and other 90 loci*	NHEJ	[174]
Maize	*ZB7, 2g332562, 2g080129, 2g099580, 2g170586, 2g438243,*	NHEJ	[175]
Maize	*ARGOS8*	NHEJ	[176]
Maize	*AGO18a, Ago18b, a1, a4*	NHEJ	[177]
Moss	*PpAPT*	NHEJ, HDR	[178]
Moss	*PpKAI2L, PpAP2/ERF*	NHEJ	[179]
*N. oceanica*	*NR*	NHEJ	[180]
*N. attenuata*	*AOC*	NHEJ	[123, 159]
*N. benthamiana*	*PDS3*	NHEJ, HDR	[96]
*N. benthamiana*	*PDS*	NHEJ	[181–183]
*N. benthamiana*	*PCNA, PDS*	NHEJ	[60]
*N. benthamiana*	*FLS2, BAK1*	NHEJ	[81]
*N. benthamiana*	*PDS, blspH*	NHEJ	[184]
*N. benthamiana*	*XT1, XT2*	NHEJ	[185]
*N. benthamiana*	*EDS1a, PAD4*	NHEJ	[161]
*N. tabacum*	*GFP (transgene)*	NHEJ	[153]
*N. tabacum*	*PDS, PDR6*	NHEJ	[186]
*N. tabacum*	*mCherry (transgene)*	NHEJ	[187]
Petunia	*PDS*	NHEJ	[188]
Petunia	*NR*	NHEJ	[189]
Populus	*4CL1, 4CL2, 4CL5*	NHEJ	[64]
Populus	*PDS*	NHEJ	[190, 191]
Potato	*IAA2*	NHEJ	[192]
Potato	*ALS*	NHEJ	[142, 193]
Potato	*GBSS*	NHEJ	[194]
Potato	*MYB44*	NHEJ	[195]
Rice	*PDS, BADH2, MPK2, 02g23823*	NHEJ	[65, 83]
Rice	*MPK5*	NHEJ	[196]
Rice	*ROC5, SPP, YSA*	NHEJ	[149, 197]
Rice	*MYB1*	NHEJ	[151, 197]
Rice	*DERF1, EPSPS, MSH1, PDS, PMS3*	NHEJ	[197]
Rice	*SWEET11*	NHEJ	[198]
Rice	*SWEET11, SWEET14*	NHEJ	[153]
Rice	*CAO1, LAZY1*	NHEJ	[199]
Rice	*BEL*	NHEJ	[200]
Rice	*SWEET11, SWEET13, SWEET1a, SWEET1b, CPS4, CYP99A2, CYP76M5, CYP76M6, KO1, KOL5*	NHEJ	[76]
Rice	*CDKA2, CDKB1, CDKB2*	NHEJ	[201]
Rice	*MPK1, MPK2, MPK5, MPK6, PDS*	NHEJ	[202]
Rice	*ALS*	HDR	[97, 98]
Rice	*GSTU, MRP15, ANP, WAXY, 7 FTL genes, and 21 other genes*	NHEJ	[80]
Rice	*AOX1a, AOX1b, AOX1c, BEL*	NHEJ	[203]
Rice	*DsRed (transgene), YSA, PDS, DL*	NHEJ	[204, 205]
Rice	*P450, DWD1*	NHEJ	[123]
Rice	*RAV2*	NHEJ	[78]
Rice	*DMC1A, DMC1B*	NHEJ	[87]
Rice	*NAL1, LPA1, LG1, GL1*-*1*	NHEJ	[206]
Rice	*DEP1, ROC5*	NHEJ	[207]
Rice	*Gn1a, DEP1, GS3, IPA1*	NHEJ	[53]
Rice	*ERF922*	NHEJ	[56]
Rice	*OST2*	NHEJ	[208]
Rice	*CSA*	NHEJ	[54]
Rice	*RUPO*	NHEJ	[209]
Rice	*EPSPS*	NHEJ, HDR	[210]
Rice	*TMS5*	NHEJ	[55]
Rice	*PMR*	NHEJ	[211]
Rice	*MEGs, PEGs*	NHEJ	[212]
Rice	*Hd2, Hd4, Hd5*	NHEJ	[213]
Rice	*SBEI, SBEIIB*	NHEJ	[214]
Rice	*ACT, GST*	HDR	[99]
Rice	*RBOHH*	NHEJ	[215]
Rice	*EPFL9*	NHEJ	[116]
Salvia miltiorrhiza	*CPS1*	NHEJ	[216]
Sorghum	*DsRED2 (transgene)*	NHEJ	[153]
Soybean	*GFP (transgene), 07g14530, 01g38150, 11g07220, miR1514, miR1509*	NHEJ	[217]
Soybean	*06g14180, 08g02290, 09g00490, 12g37050*	NHEJ	[218]
Soybean	*PDS11, PDS18*	NHEJ	[147]
Soybean	*DD20, DD43, ALS*	NHEJ, HDR	[219]
Soybean	*FEI1, FEI2, SHR, bar (transgene)*	NHEJ	[220]
Soybean	*Rj4*	NHEJ	[72]
Sweet orange	*PDS*	NHEJ	[221]
Sweet orange	*LOB1*	NHEJ	[222]
Tomato	*SHR, GFP (transgene)*	NHEJ	[223]
Tomato	*AGO,* 08g041770, 07g021170, 12g044760	NHEJ	[224]
Tomato	*RIN*	NHEJ	[225]
Tomato	*PDS, PIF4*	NHEJ	[226]
Tomato	*SIAGL6*	NHEJ	[227]
Tomato	*SP5G*	NHEJ	[228]
Tomato	*SIBOP*	NHEJ	[229]
Tomato	*SIIAA9*	NHEJ	[230]
Tomato	*MLO*	NHEJ	[231]
Wheat (common)	*MLO*	NHEJ	[7, 65]
Wheat (common)	*INOX*	NHEJ	[183]
Wheat (common)	*GASR7, GW2, DEP1, NAC2, PIN1, LOX2,*	NHEJ	[66]
Wheat (common)	*Ubi, MLO*	HDR	[100]
Wheat (Durum)	*GASR7*	NHEJ	[66]

The intrinsic property of CRISPR-Cas9 for targeting viral DNA for cleavage makes it a great tool to increase plant immunity against DNA viruses. For example, such immunity has been shown in tobacco by stably expressing Cas9 and introducing gRNAs that target geminiviruses [58]. Many similar studies have targeted geminiviruses because they must maintain circular structure for replication, thus one DSB will destroy the virus [59]. Tobacco with resistance to the geminiviruses beet severe curly top virus, bean yellow dwarf virus, and tomato yellow leaf curl virus have been created [58, 60, 61]. These findings were also replicated in Arabidopsis [61]. Because Cas9 can complex with any compatible and programmable gRNAs, it may offer a robust protection strategy against double stranded DNA viruses. Single stranded viruses can also be potentially targeted by NMCas9 which exhibit DNase H activity [62].

CRISPR-Cas9 is a valuable reverse genetic tool in plant science research. Large chromosomal deletion in Arabidopsis was used to demonstrate redundant functionality of tandem arrayed *CBF* genes in cold acclimation [63] (Fig. 3d). CRISPR-Cas9 based reverse genetics was even made possible in poplar [64], a woody tree that has traditionally proven difficult for genetic manipulation. Despite challenges with editing polyploidy plants, both hexaploid bread wheat and tetraploid durum wheat were effectively edited by CRISPR-Cas9 [7, 65, 66]. Editing of the tetraploid cotton genome was also recently reported [67, 68]. *Camelia sativa* is a hexaploid relative to Arabidopsis and editing three copies of the *FAD2* gene was demonstrated when screen was carried to T3 generation [69, 70]. Using CRISPR-Cas9, two recent studies disproved conclusions made by earlier work using traditional genetic techniques, further demonstrating that CRISPR-Cas9 is a great addition to existing genetic tools. In one study, knockout alleles of *ABP1* were generated in Arabidopsis and it was discovered this gene is not required for auxin signaling or development as originally thought [71]. In another study [72], *Rj4* was found to control nodulation specificity in soybean and the identity of this gene confirmed by CRISPR-Cas9 corrected earlier reports.

CRISPR-Cas9 will also further reverse genetic studies on non-protein coding genes (Fig. 3b) and regulatory elements (Fig. 3c). MicroRNAs are short RNAs that can repress translation, but mostly cleave mRNA transcripts [73]. Both mechanisms silence protein expression. Long non-coding RNAs are diverse groups of non-coding transcripts longer than 200 nucleotides whose function is poorly understood in plants [74]. Small indel mutations in non-protein coding genes may not alter or destroy their function, making them more challenging targets with CRISPR-Cas9 [75]. CRISPR-Cas9 mediated targeted chromosomal deletion is very efficient in rice [76] and this approach was recently applied for deleting microRNA genes in Arabidopsis [77]. Moreover, CRISPR-Cas9 was used to target a non-coding regulatory element of *OsRAV2* in rice to confirm its function in response to salt treatment [78].

## Multiplex CRISPR-Cas9 systems

One distinct advantage of CRISPR-Cas9 over TALEN is the ability to multiplex (Table 1). By expressing multiple gRNAs that independently pair with Cas9, multiple target sites can be mutated in a single cell. This multiplexing property of CRISPR-Cas9 has enabled targeted deletion of large chromosomal segments containing multiple genes in rice [76] and in Arabidopsis [63]. Simultaneous targeting of multiple genes can result in more than one improved trait in crops, and can also be used in basic research to deduce the role of each gene in a complex network.

The first toolkit to demonstrate multiplexing knockout of three Arabidopsis genes was released in 2014 [79]. Since then, several toolkits have been developed. A second toolkit was released in 2015 by Ma et al. [80], that constructed vectors using PCR and Golden Gate cloning. These constructs were validated in both monocots and dicots. A third toolkit was released in that same year by Lowder et al. [81]. This kit contains vectors that could be used for genome editing and transcriptional regulation without the need for PCR, ensuring that no mutations occur during assembly. Other multiplex systems were also developed that, while more time consuming, allowed for targeting of up to six target sites or theoretically unlimited target sites respectively [82, 83].

## Paired CRISPR-Cas9 nickase for improving editing specificity

**Fig. 4:**
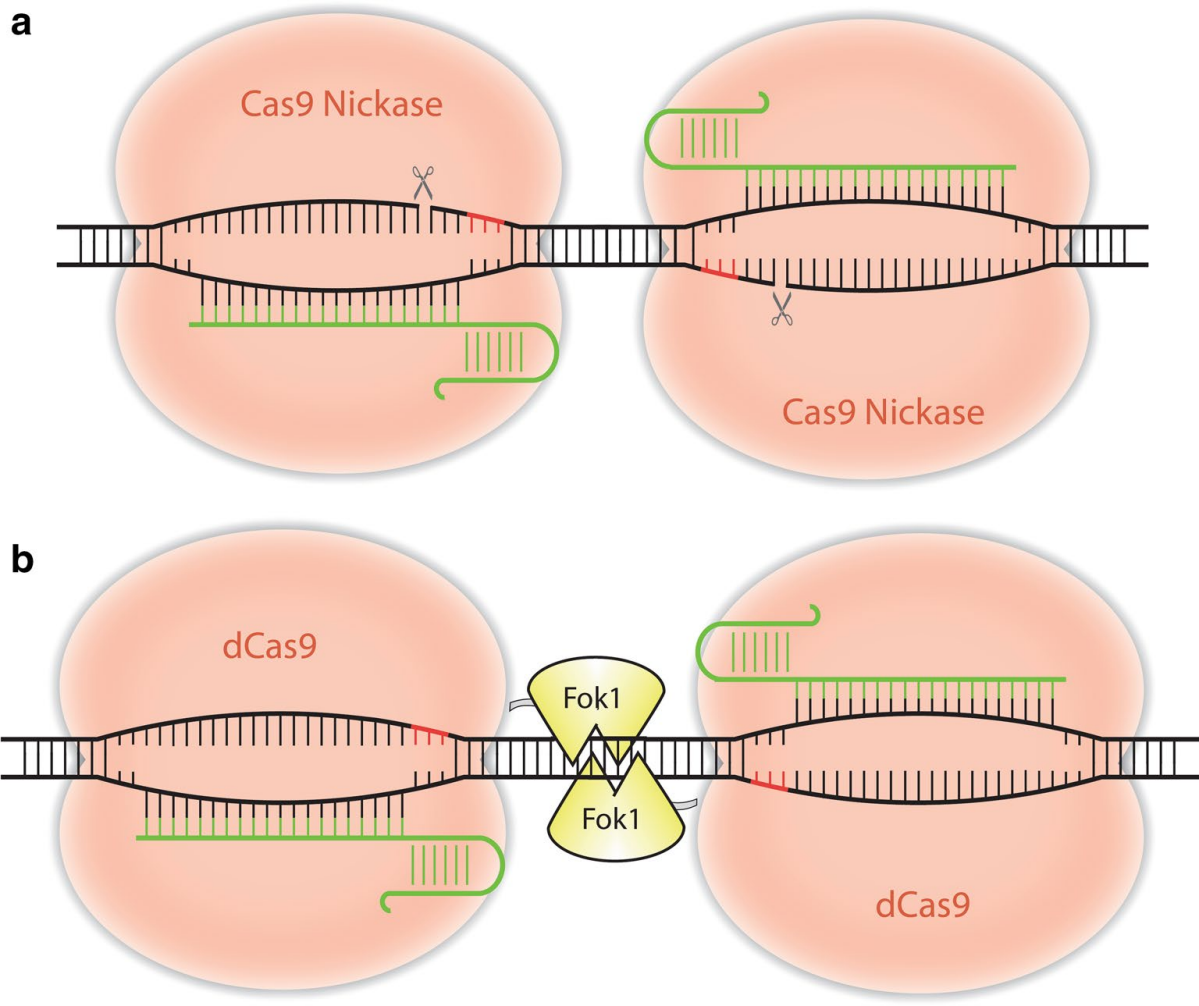
Paired Cas9 nickase and FokI-dCas9 systems. Alternative Cas9 proteins can decrease off-target effects. **a** Two nickases are required to make a double-strand break, increasing the gRNA requirement and length of target sequence. **b** A catalytically dead Cas9 is paired to a Fok1 nuclease, also resulting in an increased length of target sequence for enhanced targeting specificity

TALEN works in pairs to recognize 30 bp or even longer DNA sequences and presumably has higher targeting specificity than CRISPR-Cas9 which recognizes ~20 bp DNA sequence. However, the targeting specificity of CRISPR can be improved by using a paired nickase strategy (Fig. 4a). One of the Cas9 endonuclease domains, either HNH or RuvC-like, is inactivated to produce a Cas9 nickase that can only cut one DNA strand. By pairing two nickases and their gRNAs, the target sequence grows from ~20 to ~40 bp and specificity is drastically increased. It was shown this increase in specificity results in a 20- to 1500-fold reduction in off-target effects without a decrease in cleavage efficiency in human cells [84]. There are several examples of successful genome editing using nickases in plants [85–87]. A single transcript unit (STU) was effectively shown to express Cas9 nickase and a gRNA pair [88], in which Cas9 and two gRNAs flanked by hammerhead ribozyme sequences were expressed under a single Polymerase II promoter. The ribozyme successfully processed the single transcript, demonstrating a system for simultaneous, inducible expression of both Cas9 and gRNAs.

Alternatively, FokI-dCas9 can be engineered to work in pairs [89, 90], which relies on fusing a catalytically dead Cas9 (dCas9) with a FokI nuclease domain (Fig. 4b). When the two Fok1-dCas9s are carefully positioned on both DNA strands, the gRNAs lead dCas9 to the target sites and FokI nuclease domains dimerize resulting in DNA cleavage. As with the paired nickase strategy, the requirement of two gRNAs should decrease off-target effects. This takes advantage of the simple design of gRNAs and avoids the protein engineering required for TALEN. However, the editing frequency for both techniques will need to be improved for wide-scale adoption.

## HDR based genome editing with TALEN and CRISPR-Cas9

**Fig. 5:**
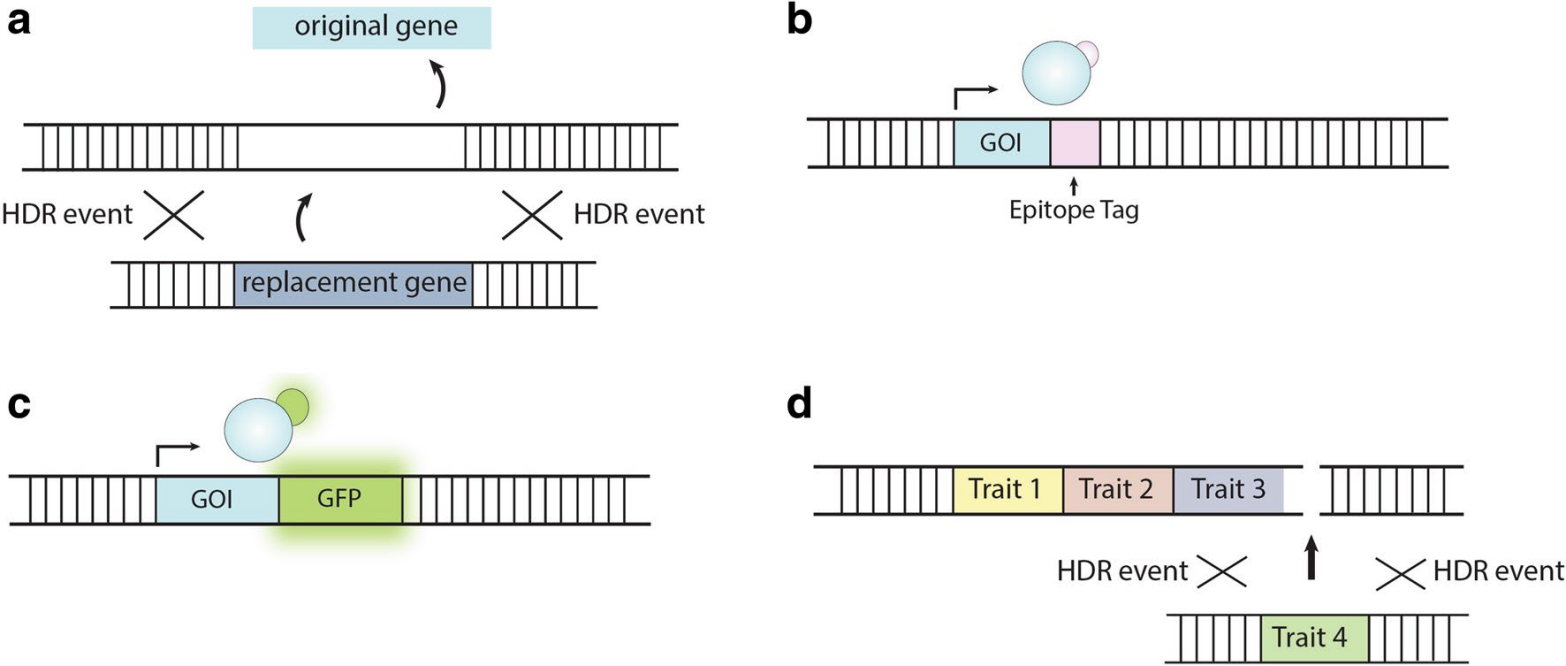
HDR based genome editing applications. **a** Gene replacement is applicable for basic research and agriculture. **b** HDR can add a tag to a protein for easy purification and study. **c** Fluorescent proteins such as green fluorescent protein (GFP) can be fused to a gene of interest for in vivo study. **d** Gene stacking is useful for placing genes physically close together on a chromosome. This is accomplished by creating a target site for HDR at the end of each gene, which allows for modular addition of genes

There are many powerful applications for HDR based genome editing using both TALEN and CRISPR-Cas9. The applications include, but are not limited to, gene replacement (Fig. 5a), epitope tagging (Fig. 5b) or florescent protein tagging (Fig. 5c) of endogenous genes, and gene insertion which can be used for trait stacking (Fig. 5d).

Gene replacement with HDR was first accomplished using TALENs in human cells in 2011 [91], but it wasn’t until 2013 that HDR initiated by TALEN was demonstrated in plants [43] (Table 2). Barley was the first monocot to demonstrate HDR with TALEN. A green fluorescent protein (GFP) was converted into yellow fluorescent protein (YFP) by one amino acid change with a 3% efficiency in protoplasts, demonstrating an effective system for optimizing TALENs [92]. Replacing *ALS* with an herbicide resistant gene was successful in tobacco protoplasts and rice with TALEN [43, 93]. In the tobacco protoplasts, about 30% of transformed cells had NHEJ mutations and 14% showed targeted insertion due to HDR [43]. For this study, transient expression of TALEN was efficient enough to get edited plants without selection. In rice, it was reported that between 1.4 and 6.3% of transformants had one or both alleles edited [93]. In tomato, targeted insertion of a strong promoter ahead of the *ANT1* gene led to ectopic accumulation of anthocyanin, producing purple tomatoes [94]. The study utilized a geminivirus replicon system that has the advantage of amplifying the genome editing reagents in plant cells [95].

HDR utilizing CRISPR-Cas9 was first demonstrated in 2013 [96] (Table 3). A plant codon-optimized Cas9 and gRNAs were transiently expressed in Arabidopsis and tobacco protoplasts for targeting respective *PDS* genes. A much higher mutagenesis frequency was observed in the tobacco protoplasts compared to Arabidopsis. HDR was accomplished at 9% frequency with a donor template harboring an *Avr*II digestion site, a 533 bp left homology arm, and a 114 bp right homology arm. This proof-of-concept study demonstrated that it is possible to replace a wild-type gene with an altered one using CRISPR-Cas9 in plant cells. A year later, germline editing of the *ADH1* gene was demonstrated in Arabidopsis [86]. CRISPR-Cas9 has also been used to alter *ALS* in rice to confer herbicide resistance [97, 98] and both studies explored different strategies to enhance HDR in rice. In one study, plants with a *lig4* mutation were shown to have between a 0.147 and 1% gene targeting efficiency and these contained biallelic mutations [98]. Lig4 is involved in the classic NHEJ pathway (Fig. 1a) and *Lig4* mutants have been shown to undergo increased rates of HDR and microhomology-based alternative NHEJ in Arabidopsis [22]. In the second study, the authors observed high frequency HDR when using two gRNAs for cutting off the target gene and liberating donors that were provided in the form of both plasmids and free double-stranded DNAs [97].

For all HDR applications, efficiency will need to be improved. Increasing the efficiency of SSN delivery will greatly help genome editing, including HDR applications. If a higher percentage of plants or plant cells can receive SSNs, then more of them will have the potential to undergo HDR without increasing sample size. Although easy to use, agrobacterium-mediated delivery is not as efficient as ballistic bombardment because the latter can introduce multiple copies of donor DNA [93, 98]. One of the potential methods that may solve issues with difficult delivery, as well donor copy number, is geminivirus delivery. In tomatoes, geminiviruses replicons were found to create mutations at a 10-fold higher frequency when compared to agrobacterium mediated transfer [94]. Recently, geminivirus systems were successfully used for CRISPR-Cas9 mediated HDR in rice [99] and wheat [100]. Alternatively, donor DNA may be liberated from integrated chromosome regions with an *in*-*planta* gene targeting strategy [86, 101]. The second issue to address is low occurrence of HDR in cells, especially in non-dividing cells. If all cells in culture or *in planta* were synchronized, then SSN and donor DNA could be introduced during replication which will boost HDR events. Cas9 nickases, with their ability to create single stand breaks (SSBs), have been utilized for HDR in Arabidopsis at high efficiencies and the authors have speculated the mechanism of HDR initiated by SSBs could be different from that of DSBs [85]. The mechanism of SSB based HDR, if discovered, should be useful for enhancing HDR. There are many exciting possibilities for HDR based genome editing, and innovative ideas will continue to further this area.

## TAL effector and CRISPR-Cas9 for transcriptional regulation

**Fig. 6:**
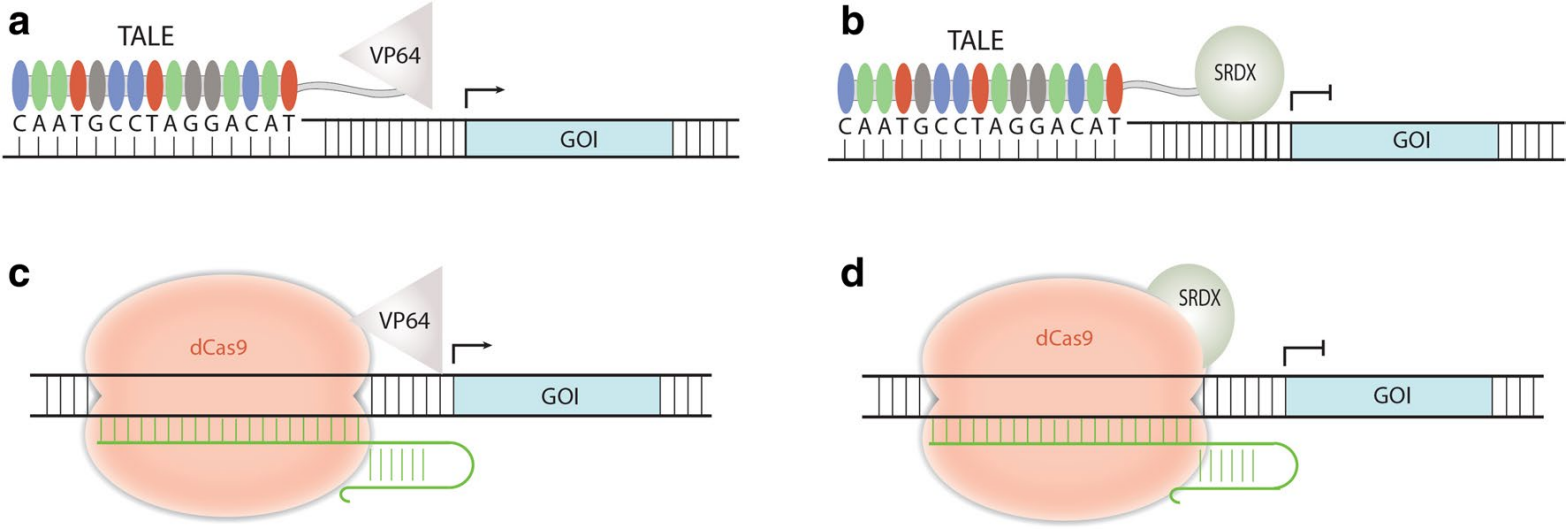
TALE and CRISPR-Cas9 based transcriptome modulation systems. **a** The activator VP64 is fused to TALE for gene activation. **b** The repressor SRDX is fused to TALE for gene repression. **c** The activator VP64 is fused to dCas9 for gene activation. **d** The repressor SRDX is fused to dCas99 for gene repression

Either a TAL effector or a deactivated Cas9 (dCas9) can be fused to an activator such as VP64 [102] or a repressor such as SRDX [103] for transcriptional regulation in plants (Fig. 6). There may be some differences intrinsic to TAL effector and Cas9 that make one more suitable for activating or repressing gene expression than the other. To date, no study has been carried out to make an accurate comparison of both systems in plants.

TAL effectors are natural transcriptional activators in plants [104, 105]. This property was cleverly used for decoding the DNA recognition code of TAL repeats [25]. Although the endogenous transcriptional activation domain of a TAL effector seems potent for activation, it could be swapped with VP64 to make smaller proteins (Fig. 6a). TAL repeats, when fused to SRDX, repressed gene expression by more than twofold in Arabidopsis [106]. Interestingly, it was recently reported in Arabidopsis that binding of TAL proteins to the sense strand of a gene of interest is enough to result in gene repression [107], which is likely due to TAL proteins blocking transcription. Despite proven concept, there is almost no report on utilizing de novo-engineered TAL activators or repressors in plant research. This could be due to the difficulty of engineering of TAL proteins and multiplexing them in plant cells.

CRISPR-Cas9 may be more suitable for developing transcriptional regulation tools due to facile engineering and multiplexing. CRISPR-dCas9 based activators and repressors were demonstrated in transiently transformed tobacco [108] and in stably transformed Arabidopsis [81]. In the latter study, a tool kit was developed for easy assembly of a final T-DNA construct for simultaneous transcriptional modulation at multiple genetic loci in plants [81]. By targeting dCas9-VP64 to a highly-methylated promoter region, a 400-fold increase in mRNA expression of the imprinted gene, *AtFIS2*, occurred in Arabidopsis rosette leaves [81]. The result demonstrated that methylated DNA, difficult to target with TAL proteins [109], is targetable by CRISPR-Cas9 (Table 1). Although these results are exciting, they merely represent the first generation of such activators and repressors. Further improvement of CRISPR-dCas9 based transcriptional regulation systems for high efficiency in plants is anticipated.

## Future perspective

CRISPR-Cas9 has been widely adopted for basic and applied research and as efficiency improves will continue as a popular tool. Currently, gene targets are somewhat limited by the NGG PAM site required by SpCas9 [38] (Table 1). However, target ranges will broaden as more systems are further explored. Orthogonal Cas9s have garnered attention for their unique PAM sites and gRNA structure, creating the possibility of expressing multiple Cas9s and gRNAs in a cell without interference. These orthogonal Cas9 variants differ in size and specificity as well as PAM sequences. Some of the most promising are NmCas9, StCas9 and SaCas9, all of which have been demonstrated in human cells [110] and the latter two in plants [111–114]. A CRISPR-Cpf1 system was reported in 2015 and it differs from the Cas9 system on several key parameters [115]. Cpf1 requires only a crRNA, making the gRNA 42 nt instead of ~100 nt for Cas9. The Cpf1 PAM is TTTN and cleavage results in 5′ overhangs distall from protospacer elements. A shorter gRNA is easier to synthesize and an overhang may improve efficiency for NHEJ based gene insertion if the insert is designed with a complementary overhang. Lastly, the location of the DSB means that any indels will likely fail to disrupt the PAM site, leaving the possibility for multiple Cpf1 targeting events and allowing a second chance for gene insertion should the first attempt fail. Reports of Cpf1 in plants have also been published recently [116–121]. The CRISPR-Cpf1 system developed by Tang et al. achieved 100% mutagenesis frequency at all target sites in rice [119], demonstrating promising applications of Cpf1 in plants.

DNA independent delivery of SSNs for plant genome editing is another trend. Development of such methods are likely motivated for use in crop improvement in regards to regulation [2]. Nucleic-acid free delivery of TALEN has been successfully accomplished [122]. This study demonstrated that delivery of pure nuclease protein into protoplasts was possible albeit at a low frequency [122]. DNA-free delivery of Cas9 was accomplished by incubating Arabidopsis, rice, tobacco, and lettuce protoplasts with Cas9/gRNA ribonucleoprotein complexes [123]. Bread wheat was shown to be amenable to genome editing based on mRNA or ribonucleoprotein delivery of CRISPR-Cas9 [66, 124]. More recently, ribonucleoprotein delivery of CRISPR-Cpf1 was also demonstrated in soybean and wild tobacco protoplasts [120].

Genome editing may be achieved without introducing DNA DSBs. DNA base editing tools based on fusing cytidine deaminase to Cas9n or dCas9 were first demonstrated in human cells [125, 126]. Encouragingly, this technology was recently shown to work in rice [127–131], Arabidopsis [132], tomato [131], maize and wheat [129]. Without question, first generation base editing tools will be further expanded, improved and applied in many other plant species soon. Finally, as genome editing moves ahead into many crop plants, improving transformation and tissue culture methods will be critical for success. A recent report of using *Baby boom* and *Wuschel* genes to improve transformation efficiency in recalcitrant monocot plants set an exciting example of this endeavor [133].

## Abbreviations

DSB: double strand break
NHEJ: non-homologous end joining
HDR: homology-directed repair
PAM: protospacer adjacent motif
ZFN: zinc-finger nuclease
TALE: transcription activator like effectors
CRISPR: clustered regularly interspaced palindromic repeats
Cas9: CRISPR associated protein 9
Cas9n: Cas9 nickase
dCas9: dead or deactivated Cas9
gRNA: guide RNA
Cpf1: CRISPR from Prevotella and Francisella 1
crRNA: CRISPR RNA
GOI: gene of interest
